# A Novel Multi-Dimensional Joint Search Method for the Compression of Medical Image Segmentation Models

**DOI:** 10.3390/jimaging10090206

**Published:** 2024-08-23

**Authors:** Yunhui Zheng, Zhiyong Wu, Fengna Ji, Lei Du, Zhenyu Yang

**Affiliations:** School of Computer Science and Technology, Shandong University of Technology, Zibo 255049, China; zyh1786225568@163.com (Y.Z.); jifengna11@163.com (F.J.); m18753913017@163.com (L.D.); yangzhenyusdzb@163.com (Z.Y.)

**Keywords:** artificial intelligence, cardiac segmentation, deep learning, pixel classification, model compression

## Abstract

Due to the excellent results achieved by transformers in computer vision, more and more scholars have introduced transformers into the field of medical image segmentation. However, the use of transformers will make the model’s parameters very large, which occupies a large amount of the computer’s resources, making them very time-consuming during training. In order to alleviate this disadvantage, this paper explores a flexible and efficient search strategy that can find the best subnet from a continuous transformer network. The method is based on a learnable and uniform L1 sparsity constraint, which contains factors that reflect the global importance of the continuous search space in different dimensions, while the search process is simple and efficient, containing a single round of training. At the same time, in order to compensate for the loss of accuracy caused by the search, a pixel classification module is introduced into the model to compensate for the loss of accuracy in the model search process. Our experiments show that the model in this paper compresses 30% of the parameters and FLOPs used, while also showing a slight increase in the accuracy of the model on the Automatic Cardiac Diagnosis Challenge (ACDC) dataset.

## 1. Introduction

Due to the robust results achieved in the field of Natural Language Processing (NLP) by the transformer [1] created by the Google team, that team then introduced a transformer [2] into the field of computer vision (CV). Although this is not the first time they have introduced a transformer into computer vision, it has become the most used model by a wide range of researchers due to its simplicity and high scalability. At present, transformers have become a central pillar of a powerful network modelling architecture that is used for various computer vision tasks, such as image classification [3,4,5,6], target detection [7,8,9], and image segmentation [10,11,12,13]. Convolutional neural networks (CNNs) are traditionally used in medical image segmentation models. Due to the excellent performance of transformers in the CV field, transformer frameworks have been gradually introduced into medical image segmentation, such as TransUNet [14], SwinUnet [15], Mamba-unet [16], etc., and have achieved excellent results, but the disadvantage is that their training and inference process requires a large amount of computational resources, especially when dealing with large-scale datasets, which restricts their application in resource-constrained devices or real-time applications.

The Visual Transformer (Vit) is not widely used in practice, mainly due to its large number of model parameters and excessive training and inference costs. Recently, a large amount of research has focused on compressing Visual Transformer networks by finding more powerful and efficient architectures for their use [17,18,19]. However, many traditional network architecture search methods are consume a lot of computer resources, such as reinforcement learning, evolutionary algorithms, Bayesian optimization, etc. Guo et al. [20] introduced the Single Path One Shot (SPOS) method, which greatly decreases the search cost compared to traditional approaches. However, it still requires evaluating thousands of sub-networks to identify the optimal sub-architecture, making the process very time-consuming; it often takes several weeks to complete.

Recently, some researchers have used the scaling parameter of Batch Normalization (BN) as an indicator of operational importance to prune a network or search for sub-networks, such as the BN-NAS proposed by Chen et al. [21], the SCP proposed by Kang [22], and so on. Although the training speed of these search methods is tens of times faster than the general SPOS, not all of the models contain a BN layer. More and more scholars have introduced transformers in medical image segmentation, but traditional transformers do not contain a BN layer, and, in addition, as transformers contain a dependence on input tokens that moves from shallow to deep layers, using such a search strategy is not always practically feasible, and the structure of the searched model is not always optimal.

Most of the models in the field of medical image segmentation do not contain BN; in order to complete the search process they can not use the scaling factor in a BN as a search metric. As such, this paper adopts an explicit soft mask as a search metric to indicate the global importance of each dimension in different modules. In this paper, we perform a joint search for the three dimensions of a transformer’s patch mechanism, Multi-Head Attention (MHSA), and Multilayer Perceptron (MLP) and design additional Microsoftable masks in different modules and add the L1-paradigm regularization to force sparsity on the masks. A tanh function is also introduced to prevent the mask values from exploding in the patch search section. In addition, the search process can cause a loss of accuracy; in order to solve this problem, a correction module is introduced at the end of the model to prevent the accuracy degradation caused by the search process.

In this paper, we propose a model search method for medical image segmentation, the DFTransUNet-slim, which is a joint search method based on sparse masks with an implicit weight-sharing mechanism for the better searching of sub-networks. There is no BN layer in this model, which is better for the transformer, and the primary benefit lies in its search efficiency, as it can leverage pre-trained parameters and conduct a rapid search using them. Another advantage is its zero-cost subnet selection and high flexibility. Compared to a Single Path One Shot (SPOS) search, which requires thousands of subnets to be evaluated on validation data, once the search is completed, the method in this paper can obtain countless subnets and determine the final structure without an additional evaluation based on the trade-off between the actual device’s need for accuracy and FLOPs. Another advantage is its ability explore more detailed architectures, such as varying dimensions across different self-attention heads. The continuity of the search space across these dimensions allows for identifying architectures with unique dimensions and configurations in different layers and modules, which consistently results in superior sub-networks compared to other approaches.

In addition, there are blurred boundaries to the tissues in medical images, making it difficult to determine their exact location and shape; medical imaging devices may introduce noise and artifacts when acquiring images, which can affect the clarity and contrast of the image and thus increase the difficulty of segmentation. Different tissues or organs may also have similar grey values or texture features in an image, making it difficult to distinguish between them. In this paper, a DF module is added, which can effectively distinguish the boundaries of different tissues from the direction vectors of the pixel points of the tissue boundaries and correct misclassified pixel points. And, for models that are large, computationally expensive, and difficult to apply in resource-constrained environments, we explored a multi-dimensional search approach that can effectively reduce the model’s size.

The main contributions of this paper are as follows:

(1) A DFTransUnet model is proposed for medical image segmentation; it is based on the TransUnet framework and capable of pixel-level segmentation.
(2) By compressing the DFTransUnet framework, a new search framework, DFTransUnet-slim, is proposed. It is able to perform efficient searches on all three modules in a transformer—its Multi-Head Attention, Multilayer Perceptron, and patch mechanism.

## 2. Related Work

Efficient model and architecture search: Model compression is a method used to reduce the resource consumption of a model by reducing the number of its parameters and its computational complexity while guaranteeing its performance. Popular compression methods include channel pruning [23,24,25], quantization and binarization [26,27,28,29], knowledge distillation [30,31,32], and structure searches [33,34,35]. Howard et al. proposed MobileNets [36], which decompose the convolutional filter into depth and pointwise convolution to reduce the parameters in the convolutional neural network. Tan et al. introduced EfficientNet [37], which explores uniform scaling across the depth, width, and resolution dimensions to improve both accuracy and efficiency. Network Slimming [24], proposed by Zhuang et al., uses the BN parameter as a measure to find the optimal substructure. Liu et al. [38] proposed Joint Pruning, a method that simultaneously searches for the optimal number of channels layer by layer, while also considering depth and resolution to achieve more precise compression. NetAdapt [39] and AMC [40] employ feedback loops or reinforcement learning techniques to determine the optimal number of channels in a CNN. Additionally, various neural architecture search (NAS) methods focus on exploring different structural operations, such as 3 × 3, 5 × 5, and 7 × 7 convolutions. For instance, SPOS builds a super-network that encompasses all possible configurations and utilizes an evolutionary search to identify sub-networks within this super-network. However, these NAS methods defined on discrete operational search spaces are difficult to generalize and struggle to deal with continuous channel number search problems.

Efficient visual transformer: Many scholars are already exploring this area; for example, Dynamic-ViT [41] employed multiple hierarchical prediction modules to assess the importance of each patch in order to find the optimal selection of dynamic patches. However, this approach to patch pruning did not enhance parameter efficiency. On the other hand, ViTAS [42] used evolutionary algorithms to search for the best architectures within a specified budget. However, their search space is discrete and predefined, which significantly restricts its applicability. GLiT [17] combines a CNN and attention mechanisms to perform an evolutionary search of global and local modules and introduces a local module to model both local and global features. BigNAS [43] introduces a single-stage approach to generate efficient sub-models by slicing weight matrices. Building on this, AutoFormer [18] showed that weight entanglement is better than defining weight matrices for each possible sub-module and searching for the best sub-network using an evolutionary algorithm. However, due to the use of evolutionary algorithms for the search, their search space is just as much restricted to discrete space. S2ViTE [19] provides end-to-end sparsity exploration for Visual Transformers using an iterative pruning and growth strategy. Its structured pruning approach, based on a loss function computed by the Taylor Expansion and a score function for the L1 paradigm, eliminates complete attention heads and Multilayer Perceptron neurons, but the complete elimination of attention heads is suboptimal, limiting the learning dynamics of the transformer. Allowing the model to determine the optimal dimension for each attention head (rather than eliminating the attention heads completely) is a better alternative to pruning the Multi-Head Attention module. Huang et al. proposed Mamba-UNet [13], a medical image segmentation network that combines Visual Mamba Blocks (VSSs) with UNet architecture, enhancing long-range dependency modelling to improve segmentation accuracy.

Medical Image Segmentation: Most of the existing models for processing medical images are based on variations or modifications of the Unet [44] framework and, although the ideas behind their modifications are all different, the general framework of their models is still u-shaped. For example, Unet++ [45] designed a dense jump connection by changing the jump connection part of Unet, and the dense jump path makes it easier to optimize semantically similar feature maps and improves the model’s segmentation accuracy; Unet3+ [46] proposed a full-scale jump connection, which combines low-level details from feature maps of different scales with high-level semantics and maximizes the use of the information of full-scale feature maps to improve its segmentation accuracy. Even more models are based on variations or modifications of the Unet framework. With the interest surrounding transformers, more and more scholars have introduced transformers in medical image segmentation; the accompanying problem is that the number of model parameters increases exponentially, which greatly hinders the process of medical image segmentation in practical applications.

## 3. Proposed Approach

### 3.1. DFTransUnet

**Figure 1:**
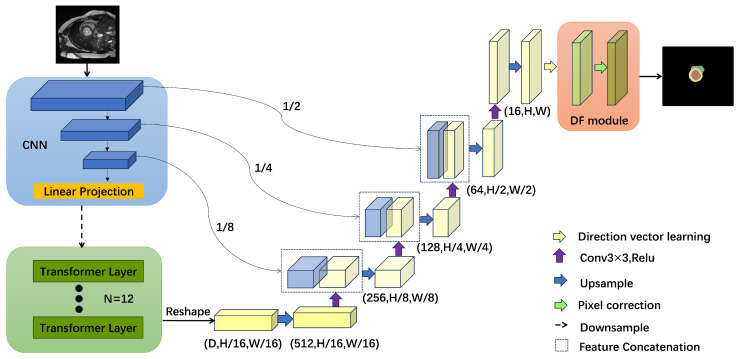
The DFTransUNet-slim model consists of four key components: the encoder, which is responsible for learning the features of the image; the decoder, which is used to recover the image’s information; the skip connection, which connects the encoder to the decoder; and the DF module, which is dedicated to correcting misclassified pixel points.

The base model used in this paper is TransUnet. The model loses accuracy to varying degrees as it performs the search, and, to compensate for this, a correction module is added to the end of the model to make up for the loss of accuracy that occurs during the search. The structure of the model is shown in Figure 1.

The pixel point classification module consists of a directional field learning module and a directional field correction module. The directional field learning module is used to learn the direction vector of each pixel point and provide it to the directional field correction module, which corrects each pixel point so as to achieve better segmentation. Specifically, given a mask with 64 channels, after 1 × 1 convolution, the directional field learning module learns the direction vector ($\vec{bm}$) of each pixel m in the mask to the nearest pixel b at the heart tissue boundary, and this learned direction vector implies rich information, such as the boundaries, shapes, and so on, of the object to be segmented. After that, the direction vectors are normalized so that the non-masked pixels are set to (0,0), and finally a two-channel direction field is obtained. The direction field can be represented by the following equation:

(1) $$D\left(m\right)=\left\{\begin{array}{cc} \frac{\vec{bm}}{\left|\vec{bm}\right|},\; & b\in mask\\ \left(0,0\right),\; & otherwise \end{array}\right.$$

The misclassified pixel points are corrected by the F module using the direction field from the D module. Initially, pixel points with vector lengths exceeding a specific threshold are eliminated as substitute pixel points. Based on the direction vectors of alternative pixels in the D module, the software selects one of eight pixels surrounding the alternative pixel and continues its iterative search for alternative pixels. This process continues until the software reaches the central region of the heart tissue. This method of choosing alternate pixel points is based on the direction vectors found in the orientation field. These vectors denote the direction from the boundary towards the central region. Utilizing these direction vectors, the erroneously classified pixel points are corrected by shifting them in the right direction, thus leading to pixel point correction. The initial mask image, $F\in {ℜ}^{\left(C\times H\times W\right)}$, is iteratively improved under the guidance of the direction field $D\in {ℜ}^{\left(2\times H\times W\right)}$ using bilinear interpolation, ultimately resulting in a more accurate mask $F^{N^{\prime }}\in {ℜ}^{C\times H\times W}$. The entire process is as follows:

(2) $$\forall P\in \Omega ,F^{i}\left(p\right)=F^{i-1}\left(p_{x}+D{\left(p\right)}_{x},p_{y}+D{\left(p\right)}_{y}\right)$$

where 1≤i≤N represents the current iteration step, N is typically set to 5, and $p_{x}$ and $p_{y}$ represent the coordinates of the pixel p. To achieve the ultimate cardiac segmentation outcome, it is necessary to combine $F^{N}$ and $F^{N^{\prime }}$ and subsequently utilize the final classifier on the merged feature maps.

### 3.2. DFTransUnet-Slim

#### 3.2.1. Achieving a Continuous Search Space

The one-shot NAS approach for CNNs explicitly defines multiple decoupled candidate blocks for each layer to train a centralized super-network. This strategy is suitable for CNNs because the candidate blocks at each layer come from a variety of sub-architectures, thus allowing for the characterization of multiple lookalike networks sampled from the super-network to be maintained during the search process. This approach does not apply to transformers, which are composed of multiple fully connected layers arranged in varying configurations across different blocks. The core components of transformers are these fully connected layers, and the search space can be expanded by sharing weights between them. This forms the basis of the search methodology employed in this paper.

For a fully connected layer with a given input dimension $D_{in}$, the conventional method involves generating multiple candidate layers within a predefined search space and then utilizing an appropriate search algorithm to identify the optimal layer from them. However, this approach has two drawbacks: (1) the search time is long, as each candidate layer needs to be at least partially trained in order to be searched, and (2) the weight matrix of each candidate layer leads to a large memory footprint. In this paper, we propose sharing the weights among all possible candidate layers to solve these problems. The maximum allowable output dimension is fixed to $D_{max}$ and a super-weight matrix $W_{\sup}\in R^{D_{\mathrm{in}}\times D_{\max}}$ is defined, and the weights of the candidate layers can be obtained by slicing from $W_{sup}$.

A single-stage search approach is utilized to establish a continuous search space by ranking the significance of each dimension within the super-weighted network. The method used in this paper can be performed directly on a pre-trained network, eliminating the need to train a super-network, and defines a mask $z\in R^{D_{\max}}$ for each dimension to be searched. The mask values, representing the importance score of each dimension, are initialized at 1 and incorporated into the model when loading the pre-trained weights. The search algorithm uses a loss function that combines cross-entropy with the L1 norm of the masks. This combination encourages the mask values to approach 0 while simultaneously minimizing the objective loss function. In this way, the optimization process implicitly drives the masks to be ranked according to their impact on the final performance of the model.

#### 3.2.2. Identifying the Optimal Search Space

The most important part of a ViT is the encoder, which consists of an MHSA and MLP, and we performed a joint search on these two modules to compress the network. For the MHSA, assume that the model has L MHSA modules, each with a maximum number of allowed heads of H. This gives a total of L × H unique attention heads. If the maximum allowed feature dimension size is fixed to d, then the size of the equivalent search space is ${\left(d+1\right)}^{L\times H}$. Searching in such a large search space is computationally very difficult; however, there are several advantages of such a diverse search space: (1) The search algorithm provides increased flexibility in adjusting the SuperTransformer to more compact architectures without sacrificing performance. (2) It improves efficiency by reducing the feature dimensions of the least important attention heads to zero, which significantly cuts down on FLOPs. As a result, the full ${\left(d+1\right)}^{L\times H}$ search space is employed in this study.

In the context of the MLP (Multi-Layer Perceptron), there are *L* modules in the entire network, with each module maintaining a fixed feature dimension size of *M*. This results in a search space of ${\left(M+1\right)}^{L}$. Integrating the MHSA and MLP within a single search algorithm is both simple and effective, as it yields a more varied range of sub-networks compared to those generated by existing approaches. Additionally, the MHSA aggregates patches, which leads to an exponential increase in the cosine similarity between the patches layer by layer, up to 0.9 in the final layer. This opens up the possibility of eliminating a large number of deeper patches and some unimportant shallow patches. Combining patch selection with a joint MHSA and MLP search allows us to extract more efficient architectures with the same number of parameters, further reducing FLOPs. Intuitively, each patch should correspond to the input image one by one, reflecting the importance of different regions in the image; as such, dynamic patch selection is more effective.

#### 3.2.3. Single Search with L1-Sparsity

The search method used in this paper is mainly based on the effect of the mask value on the final performance of the ranking; once the ranking is completed, the dimension with the lowest mask value will be eliminated. Let $f:R^{x}\to R^{y}$ denote a ViT network, which converts input x to target output y through the weights W and intermediate activation vectors $T\in R^{d}$, where T is constructed by the weights W and input x together. In addition, a set of sparse masks $z\in R^{d}$ is defined, where $z_{i}\in z$ refers to the mask associated with the intermediate vectors $t_{i}\in T$, and multiplying $z_{i}$ with the corresponding $t_{i}$ is able to apply the masks to the transformer. The overall optimization objective can be expressed by the following equation:

(3) $$\min_{\left(w,z\right)}\left(L_{\mathrm{CE}}\left(f(z\cdot T(W,x)),y\right)+{∥z∥}_{l}\right)$$

A uniform mask is used to search for the optimal dimension size of the head, the dimension size of the MLP, and the patch in the MHSA. In a transformer network with L-layer Multi-Head Attention and Multilayer Perceptron blocks, each block consists of H attention heads and the input tensor of each Multi-Head Attention layer is $t_{\mathrm{al}}\in R^{N\times D}$, where N is the number of patches and D is the global feature dimension. Inside the attention head i of each Multi-Head Attention module, $t_{a}l$ is transformed into $q_{i}\in R^{N\times d}$, $k_{i}\in R^{N\times d}$ and $v_{i}\in R^{N\times d}$ via the fully connected layer, where d denotes the feature dimension of each attention head. Let the vectors corresponding to the masks in the Lth layer and H head be $z_{\mathrm{al},h}\in R^{d}$; the mask vectors can be masked to act on the Multi-Head Attention module by multiplying them with $q_{i}$, $k_{i}$, and $v_{i}$, and the total likelihood of the Multi-Head Attention module that can be explored is ${\left(d+1\right)}^{L\times H}$. The internal computation process of a Multi-Head Attention module with sparse masking is as follows:

(4) $$A_{i}=\mathrm{softmax}\left(\frac{\left(q_{i}\cdot z_{\mathrm{al},h}\right){\left(k_{i}\cdot z_{\mathrm{al},h}\right)}^{T}}{\sqrt{d}}\right)$$

(5) $$O_{i}=A_{i}\cdot \left(v_{i}\cdot z_{\mathrm{al},h}\right)$$

(6) $$t_{\mathrm{ml}}=\mathrm{projection}\left(\left[O_{1},\ldots ,O_{H}\right]\right)$$

where $t_{\mathrm{ml}}\in R^{N\times D}$ is the output of the multi-headed attentive block, which subsequently becomes the input of the Multilayer Perceptron block. Inside the Multilayer Perceptron block, $t_{m}l$ is projected to a higher dimensional space through a fully connected layer $f_{1}$. The $t_{m}l$ projection results in an intermediate vector $t_{\mathrm{el}}\in R^{N\times M}$ which is then projected back to $R^{N\times D}$ again through another fully connected layer $f_{2}$. We define the vector $z_{\mathrm{ml}}\in R^{M}$ corresponding to the mask in its layer l and add the mask to the Multilayer Perceptron by multiplying it with $t_{e}l$, and thus the total possibilities of Multilayer Perceptron modules that can be explored are ${\left(M+1\right)}^{L}$. The following equations show the computation of the mask with the Multilayer Perceptron modules:

(7) $$t_{\mathrm{el}}=f_{1}\left(t_{\mathrm{ml}}\right)\cdot z_{\mathrm{ml}},\;t_{\mathrm{al}+1}=f_{2}\left(t_{\mathrm{el}}\right)$$

For patch selection, different mask values corresponding to each patch in each layer are made and patches with lower mask values are eliminated. Because of the global single search used, occasional anomalies can occur where a patch is removed in a shallower layer but remains in a deeper one. However, the rarity of such occurrences indicates that the L1 sparsity-based search strategy effectively matches patches one-to-one based on their importance, and the patches eliminated in shallow layers implicitly force their deeper counterparts to become less important and hence eliminated. To cope with this limited number of anomalous patches, once a patch is eliminated in an earlier layer, it is also eliminated in subsequent layers while imposing a budget. Additionally, applying the tanh activation function to a patch-specific mask before performing the dot product with the patch prevents the mask values from exploding.

Once the ranking is complete, low-ranked dimensions or patches in the network are removed based on the target budget. For joint searches that include a Multi-Head Attention module and Multilayer Perceptron, the budget can be roughly converted into FLOPs and parameter limits due to the linear correlation between the number of dimensions and both the FLOPs and parameters. Additionally, the inclusion of patch selection further reduces the FLOPs. This approach allows for the transition of weights from continuous masks in the search phase to binary or null masks in the final architecture.

The overall process of the algorithm is as follows (Algorithm 1):
**Algorithm 1** Algorithm process framework**Input:** Original ViT Model, Target Budget;**Output:** Compressed and Optimized ViT Model
  1:Define Search Space: Select the parts of the model to optimize;  2:Apply Sparsity Constraint: Introduce sparsity to reduce unimportant parameters;  3:Multi-Dimensional Search and Evaluation: Conduct a search within the defined space and evaluate candidate models based on the target budget;  4:Choose the best sub-model and fine-tune it for optimal performance;  5:Determine the Optimal Model: Identify the final compressed and optimized model;


## 4. Experiments

### 4.1. Dataset: Automatic Cardiac Diagnosis Challenge

For the sake of a fair experiment, this paper utilized the publicly accessible dataset of the 2017 Automated Cardiac Diagnostics Challenge (ACDC). This dataset includes scans from 100 actual patients from the Dijon University Hospital. Each image undergoes modification and labelling by two highly experienced clinical experts with over a decade of professional experience. These annotations include annotations for the left ventricle (LV), right ventricle (RV), and myocardium (MYO). These MR images have been evenly divided into five subgroups, including normal cases, heart failure with infarction, dilated cardiomyopathy, hypertrophic cardiomyopathy, and right ventricular abnormality. The images utilized in our model are two-dimensional, while the ACDC dataset consists of three-dimensional images. In order to facilitate these experiments, all 3D images were sliced from the bottom of the ventricle to the top of the heart. Furthermore, the dimensions of the slices were less than 10 mm. Their spatial resolution within the short-axis plane varies between 0.83 and 1.75 mm^2^ per pixel. We have randomly split these 100 cases in a 7:1:2 ratio, with 70% used for training, 10% for validation, and 20% for testing.

### 4.2. Evaluation Metrics

In these experiments, we used the Dice Similarity Coefficient (DSC), which is a widely utilized metric in medical image segmentation research for assessing model performance. The DSC is a common widely utilized similarity measure function for sets, typically employed to calculate the similarity between two samples:

(8) $$DSC\left(m_{t},m_{p}\right)=\frac{2|m_{t}⋂m_{p}|}{|m_{t}|+|m_{p}|}$$

where $m_{t}$ indicates the real result and $m_{p}$ indicates the predicted result.

### 4.3. Implementation Details

The workflow of the DFTransUNet-slim framework in this paper consists of three steps, which are as follows: (1) The model is initialized with pre-trained weights, sparsity masks are introduced, and both are co-trained for 50 cycles using AdamW with a set learning rate and weight decay. (2) Budget selection, which involves sorting the masks based on their values after the search process is complete. According to the target compression budget, the dimensions with lower rankings are eliminated from the super-network, resulting in the extraction of the final model structure. (3) Retraining: finally, the extracted compressed structure is retrained for 50 cycles with the same settings based on the original pre-training settings. All the following experimental results are tested on the ACDC dataset.

Unidimensional Search: In order to test the effect of the Multi-Head Attention and multi-layer perceptron modules on the final model’s performance, and to introduce sparsity masks in the corresponding modules of the DFTransUnet-slim, the two super-networks were searched separately, with the sparsity weights of the Multilayer Perceptron and Multi-Head Attention modules’ dimensions being 1 × $10^{-4}$. The number of post-search model parameters with different budgets, the FLOPs, and the final accuracies, and all the results in the experiments are the result of retraining after the training is completed. Under the same budget, the multi-head self-attentive search outperforms the Multilayer Perceptron search, but the Multilayer Perceptron performs better in terms of parameter and FLOP compression (Table 1).

Parameter Search: Next, the weights corresponding to each module of the Multi-Head Attention and Multilayer Perceptron searches are jointly searched in the two super-networks, starting with equal sparsity weights of 1 × $10^{-4}$, with a comprehensive search performed to achieve the best performance. The results are shown in Table 2. In this table, W1 and W2 represent the weights corresponding to the Multi-Head Attention and Multilayer Perceptron modules, respectively.

Unified search: Then the MHSA and MLP were searched in a unified way and the final results are shown in Table 3. From the results, it can be seen that as the budget decreases, the number of parameters and FLOPs also decrease, along with the accuracy of the model.

Multi-Dimensional Joint Search: Finally, a joint search is conducted across all dimensions—the MHSA, MLP, and patch selection—using optimal sparsity weights for the MHSA and MLP (2 × $10^{-4}$ and 4 × $10^{-5}$, respectively) for patch sparsity. The results are presented in Table 4. These experiments use an optimal patch sparsity weight of 1 × $10^{-4}$ and, to demonstrate the impact of patch selection under varying budgets, the MHSA and MLP budgets are fixed at 70%, with the results of this also detailed in Table 4.

Since the process of searching leads to more or less accuracy degradation, this paper adds an additional DF module to compensate for the accuracy degradation caused by searching and explores the effect of this DF enhancement under different patch budgets, with the results shown in Table 5. The hyperparameters used in these experiments are the same as those used in Table 4. The results show a slight increase in the number of parameters in the model after adding the DF module again, and the accuracies are all higher, but the accuracy is lower than that of the original model when the budget is 50%. Therefore, a budget of 60% is finally used throughout the rest of this paper.

**Figure 2:**
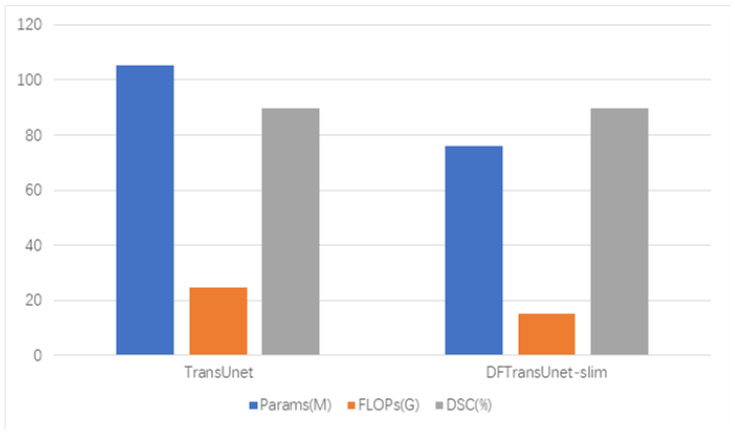
The results in terms of the number of parameters, FLOPs, and accuracy are compared with the original TransUnet model.

Ultimately, the search framework proposed in this paper compresses the number of parameters in the model by nearly 30%, and the accuracy of the model not only does not decrease, but also slightly improves; the results are shown in Figure 2.

**Figure 3:**
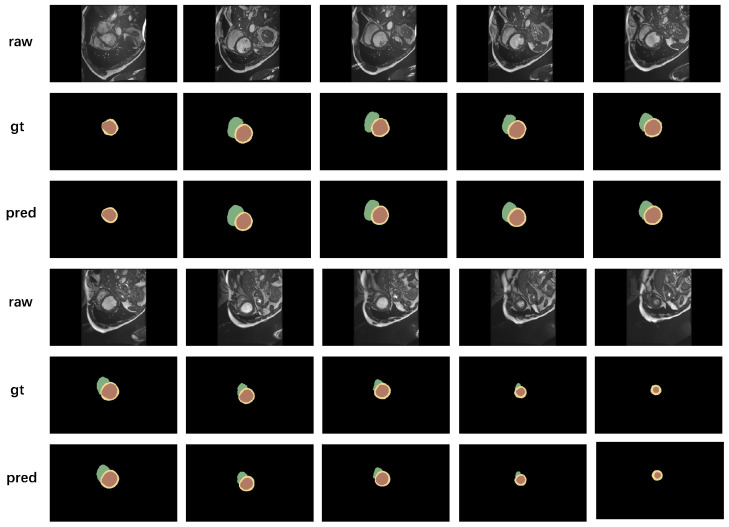
The figures show a comparison of different slices from the same patient, including the original images, manual segmentation results, and our predicted results. From top to bottom, they represent the original image (raw), manual segmentation results (gt), and predicted results (pred).

As shown in Figure 3, we selected a complete 3D MRI image of a real patient from the ACDC dataset and segmented it into 10 2D slices. In these images, both the true segmentation outcomes and those produced by our model are presented. There is almost no discernible difference between the two, which demonstrates the outstanding performance of our model in the task of medical image segmentation.

## 5. Conclusions

In this paper, we propose a flexible and efficient subnet discovery search strategy that enables medical image segmentation by implementing model sparsity in a ViT. The proposed method can perform an end-to-end joint search on three dimensions of the ViT, including its patch mechanism and Multi-Head Attention and Multilayer Perceptron modules. The global importance factor is crucial and additional differentiable soft masks are designed for different modules to reflect the importance of each dimension, in addition to forcing mask sparsification during the search process to achieve L1 sparsity.

## Figures and Tables

**Table 1:** Exploration of the impact of both the MSHA and MLP on the model. The budget indicates the % of active dimensions of their respective search modules across the network.

Budget	Module	Params (M)	FLOPs (G)	DSC (%)
-	-	105.1	24.80	89.71
70	MHSA	95.1	22.09	89.65
60		91.7	21.01	89.44
50		88.3	19.93	89.22
40		85.0	18.84	89.16
70	MLP	85.5	20.48	89.59
60		78.2	18.67	89.32
50		71.6	17.23	89.18
40		64.3	15.62	89.03

**Table 2:** Exploration of the weights corresponding to both the MHSA and MLP. W1 and W2 represent the weights corresponding to the MHSA and MLP, respectively.

W1	W2	DSC (%)
1 × $10^{-4}$	1 × $10^{-4}$	89.59
2 × $10^{-4}$	1 × $10^{-4}$	89.52
2 × $10^{-4}$	2 × $10^{-4}$	89.56
2 × $10^{-4}$	3 × $10^{-4}$	89.59
2 × $10^{-4}$	4 × $10^{-4}$	89.63
2 × $10^{-4}$	5 × $10^{-4}$	89.60
3 × $10^{-4}$	1 × $10^{-4}$	89.45
4 × $10^{-4}$	1 × $10^{-4}$	89.37

**Table 3:** A unified search is performed of the MHSA and MLP.

MLP	MHSA	Params (M)	FLOPs (G)	DSC (%)
-	-	105.1	24.8	89.71
80	80	84.6	19.94	89.67
70	70	75.5	17.79	89.63
60	60	67.8	15.73	89.52
50	50	59.5	14.68	89.23

**Table 4:** A joint search performed on all dimensions (MHSA, MLP, and patch selection).

Budget	Params (M)	FLOPs (G)	DSC (%)
-	75.5	17.79	89.63
80	75.5	16.71	89.47
70	75.5	15.63	89.38
60	75.5	15.09	89.26
50	75.5	13.67	89.13

**Table 5:** Comparison of the effect on the model after adding the DF module.

Budget	Without DF		With DF	
	Params (M)	DSC (%)	Params (M)	DSC (%)
-	75.5	89.63	76.1	90.24
80	75.5	89.47	76.1	90.11
70	75.5	89.38	76.1	89.95
60	75.5	89.26	76.1	89.81
50	75.5	89.13	76.1	89.67
